# Sir James Y. Simpson

**Published:** 1870-07

**Authors:** 


### EDITORIAL DEPARTMENT.

---

*Sir James Y. Simpson.*—While the literary world is grieving over the loss of Charles Dickens, the medical is mourning over that of one of the greatest men of the age, the news of whose death sent a thrill of regret over every part of the globe where medicine is scientifically practiced.

We glean the following sketch of the life of Dr. Simpson from European exchanges:

Prof. Simpson was born at Bathgate, in 1811, of respectable but by no means wealthy parents, he raised himself entirely by his own exertions to the position of highest eminence in his Profession.

The system of the Scottish Universities rendering education there more accessible than in the sister institutions in England, it is not unusual for the sons of the better class tradesmen to be sent to complete their schooling by attendance on some of the university courses in Edinburgh or Glasgow. In this way young Simpson, aided in a pecuniary sense by his elder brother, was sent to the University of Edingburgh, and became a member of the humanity class there, under Professor Pillans. The venerable Professor Pillans always believed that his counsel and encouragement at this time had greatly influenced Simpson's subsequent career, and he used to relate with great unction how, impressed with the versatility of his pupils acquirements, he had urged him to compete for a Stewart bursary, a scholarship which carried money with it, and being tenable for theer years, helped the holder to continue his studies in the University. To succeed required extended study of classics, which might well have deterred an aspirant from a country school, but Simpson carried off the bursary, and eventually became a student of medicine

He took his M.D. degree in 1842, and became assistant to Professor Thompson in the chair of Pathology. Thus he became familiar with a branch of scientific Medicine which constitutes the very foundation of sound Medical practice. He lectured sometimes in place of the Professor, and even then gave indicatons of originality, and of a desire to advance beyond the then received teachings on a subject as yet in its infancy.

Soon after this he began practice, and became a lecturer on Midwifery in the extra-Academical School of Edinburgh. He continued as an extra-academical lecturer for three years, when, in 1840, he was elected Professor of Midwifery in the University of Edinburgh after a severe competition. Some sort of idea may be formed of his perseverance and energy at this time by looking over the testimonials he offered to the patrons of the university as a candidate for the chair. The whole constitutes an octavo volume of more than 200 pages, and in it are to be found certificates of recommendations from all the principal Professors and teachers both in this country and on the Continent, showing that, even at this early period of his career, his reputation was established. In the same volume is a catalogue of his private museum, formed, he states, in the short space of three years, which occupies more than sixty pages, and consists of nearly 700 obstetric preparations.

At home, his consulting rooms were crowded with patients who had heard of his fame, and who came from all quarters of the world to consult him. Medical men came from every country eager to see something of his practice, and to have the pleasure of an interview with so celebrated a man.

It was possible to meet Sir James without being impressed with the diversity of his attainments and his almost universal knowledge. His memory was prodigious. It was remarked he was always the first to know about all new scientific inventions, and that he was in advance of his neighbors in information on all sorts of problems, political, scientific, or religious. To notice all his Professional works would take more space than the limits of this brief memoir can afford.

The great feature in all his contributions is their originality and wonderful lucidity of exprcssion. The most ordinary topics of Medical discussion shone out in a new light, when illumined by his touch, and matters of apparently trifling significance assumed an unsuspected importance under his treatment. He was constantly breaking new ground, and in doing so often ran counter to former notions and old prejudices.

But it is the introduction of chloroform as an agent for allaying the pangs of parturition and abolishing the pain of operations, Surgical and obstetrical, that will render Simpson's name most famous in public estimation hereafter, and rank him as equal with Harvey, Hunter, and Jenner. His other efforts and successes for the benefit of humanity can only be vaguely mentioned in ordinary conversation; this great boon can be understood and appreciated by all. Who can compute the sum of mortal agony which this single discovery has been the means of erasing from the lot of humanity?

Hearing of Morton's and Jackson's experiments with sulphuric ether in America, Simpson deliberately set himself to find some other agent which might be free from the disadvantages of ether. Taking his life in his hand, he fearlessly tried the experiment on himself of inhaling various substances, likely and unlikely, until, after various perils and disappointments, the discovery of chloroform was made. What in ancient or modern times, has been more striking as a scientific discovery or a greater boon to mankind?

Sir James was not withont recognition of his great merits during his lifetime. For his discovery of chloroform he was, in 1853, elected a Foreign Associate of the French Academy of Medicine, and in 1856 he received from the French Academy of Science the Monthyon prize of 2000 fr. "for the most important benefit done to humanity." For the same cause he received the Royal Order of Knighthood of St. Olaf from the late King of Sweden. In 1866 he was created a baronet by her Majesty Queen Victoria, and, in the same year, he received the D. C. L. of Oxford.

In 1869 the freedom of the city of Edinburgh was conferred upon him as a special honour by his fellow citizens. He was President of the Royal College of Physicians in Edinburgh in 1849, of the Medico Chirurgical Society in 1852; he held the appointment of Physician Accoucheur to the Queen in Scotland; was Foreign Associate of the Academies of Medicine in Belgium and New York, Hon. Fellow of the King's and Queen's College of Physicians, Ireland, and member of a legion of other learned and scientific societies.

The immediate cause of Prof. Simpson's death was angina pectoris, associated with heart disease. He sufferen severely from paroxysms repeated from time to time for some weeks previous to his death, and bore the pain wiih exemplary fortitude.

